# Physician-Assisted Suicide in Dementia: Paradoxes, Pitfalls and the Need for Prudence

**DOI:** 10.3389/fsoc.2021.815233

**Published:** 2021-12-22

**Authors:** Ravi Philip Rajkumar

**Affiliations:** Department of Psychiatry, Jawaharlal Institute of Postgraduate Medical Education and Research (JIPMER), Puducherry, India

**Keywords:** dementia, assisted suicide, culture, healthcare economics, ethics, religion

## Abstract

There has been an increasing drive towards the legalization of physician-assisted suicide (PAS) in patients with dementia, particularly in patients with advanced disease and severe cognitive impairment. Advocacy for this position is often based on utilitarian philosophical principles, on appeals to the quality of life of the patient and their caregiver(s), or on economic constraints faced by caregivers as well as healthcare systems. In this paper, two lines of evidence against this position are presented. First, data on attitudes towards euthanasia for twenty-eight countries, obtained from the World Values Survey, is analyzed. An examination of this data shows that, paradoxically, positive attitudes towards this procedure are found in more economically advanced countries, and are strongly associated with specific cultural factors. Second, the literature on existing attitudes towards PAS in cases of dementia, along with ethical arguments for and against the practice, is reviewed and specific hazards for patients, caregivers and healthcare professionals are identified. On the basis of these findings, the author suggests that the practice of PAS in dementia is not one that can be widely or safely endorsed, on both cultural and ethical grounds. Instead, the medical field should work in collaboration with governmental, social welfare and patient advocacy services to ensure optimal physical, emotional and financial support to this group of patients and their caregivers.

## Introduction

In recent times, euthanasia and physician-assisted suicide for specific medical conditions have been legalized in specific countries and territories (Pereira, 2011; Tomlinson and Stott, 2015). Though these terms overlap significantly, understanding the differences between them is a prerequisite for any discussion of the practices they describe (Vilela and Caramelli, 2009). According to the European Association of Palliative Care (EAPC)’s Ethics Task Force, “assisted dying” is an umbrella term that encompasses both “euthanasia” and “physician-assisted suicide.” “Euthanasia” refers to an active intervention by the physician, involving the killing of the patient by the intentional administration of drugs. On the other hand, “assisted suicide” or “physician-assisted suicide” (PAS) refers to an act in which the physician provides lethal drugs to a patient or caregiver, which are then self-administered (Materstvedt et al., 2003). In some countries, the term “medical assistance in dying” is used as a synonym for “assisted dying.” Thus, both euthanasia and PAS require the intervention of a physician, with the only difference between the two practices being the person who administers the drugs in question. The legalization of assisted dying originally occurred in the context of terminal illnesses in which recovery was considered to be impossible or extremely unlikely, and particularly in patients with severe and intractable pain or other distressing symptoms (Chambaere et al., 2010). A recent review of attitudes and practices associated with euthanasia and PAS has found this to still be the case, with 70% of cases involving patients with advanced cancer (Emanuel et al., 2016). However, in more recent times, there have been appeals to extend this practice to patients with other diagnoses, including dementia (Mondragón et al., 2019) and chronic depression or chronic pain disorders (Dees et al., 2011). In the case of dementia, arguments in favour of PAS generally center on five broad themes (Tomlinson et al., 2015; Jakhar et al., 2020):

• The economic burden posed by dementia, both at the level of individual caregivers and for society in general
• The burden faced by caregivers in terms of stress, depression, time and effort needed to perform activities of daily living for the patient, and family conflicts
• The distressing behavioural and psychological symptoms of dementia (BPSD) exhibited by several patients with these disorders, which often do not respond adequately to existing treatments. BPSD cause significant suffering to both patients and caregivers.
• Specific issues related to severe or advanced dementia, such as shortened life expectancy, poor food intake, incontinence or fluctuating levels of consciousness, and the risk of medical complications such as pneumonia.
• The perceived right of an individual to make decisions about their own life and death, particularly when cognitive and neurological impairment leads to significant suffering and loss of autonomy or identity.

Requests for PAS in patients with dementia have been gradually increasing in countries where assisted dying is legal: a recent survey of Dutch general practitioners found that nearly 42% had received such requests from patients or relatives (Schuurmans et al., 2021). Though such findings currently apply to only a small number of high-income countries, there is a strong possibility that such practices may be considered in low- and middle-income countries, particularly in those where improved healthcare has led to increases in life expectancy and in the number of elderly adults diagnosed with dementia (Mukhopadhyay and Banerjee, 2021). Though some authors have responded to such proposals with a cautious and qualified acceptance, they have also highlighted the ambiguities and ethical dilemmas inherent in such proposals (Deodhar, 2016; Jakhar et al., 2020; Mukhopadhyay and Banerjee, 2021). Moreover, attitudes towards PAS in dementia are not uniformly positive even in countries where it is legal; rather, they vary according to particular psychological, cultural, religious and economic factors (Rapp, 2016; Karumathil and Tripathi, 20202020).

It is the purpose of this article to add to this debate surrounding this topic in two ways: first, by highlighting certain inherent paradoxes in global attitudes towards assisted dying, and second, by identifying the key areas of concern regarding the implementation of such policies, from the perspectives of caregivers, healthcare professionals and wider social structures, in the specific case of dementia. The first of these goals will be addressed through an analysis of existing survey data, while the second will be addressed through a narrative review and critical analysis of the existing literature on euthanasia or PAS in patients with dementia.

## Paradoxical Correlates of Attitudes Towards PAS: Analyzing the Results of the World Values Survey

The World Values Survey, a global research project that collects information on values, beliefs and attitudes from different parts of the world and analyzes changes in these parameters over time, collected information on attitudes towards euthanasia for all causes, across 28 countries, in the period 2014–2018 (World Values Survey, 2021). In this survey, attitudes towards euthanasia in population samples from these countries were assessed by asking participants whether this practice should be legal in all cases, in selected cases, or never. As the focus of the current paper was on attitudes towards assisted dying in selected cases, the percentage of respondents for “in selected cases” (henceforth abbreviated EU-SELECT) was selected as the outcome (dependent) variable. The specific question posed to survey respondents was “please tell me whether you think euthanasia can always be justified, never be justified, or something in between.” In all countries, only participants aged 18 and above, of both sexes, were sampled. A total of 43,686 responses were received to this query. Individual sample sizes from each country ranging from a minimum of 841 (New Zealand) to a maximum of 3,531 (South Africa).

The following were included as potential predictors of attitudes towards euthanasia and were considered independent variables:

• Demographic indicators: Age and gender can crucially influence attitudes towards euthanasia. As information on the mean age and gender distribution of the study samples from each country was not available in the World Values Survey data set, two surrogate markers were used instead: average national life expectancy at birth, and proportion of women per 100 population in each country. Data on both these variables was obtained from the World Bank database (2018) (Inglehart et al., 2021).
• Indicators of economic development: Gross national income (GNI) per capital for the year 2019; Gini coefficient of economic inequality, updated for the year 2018, obtained from the World Bank database (Inglehart et al., 2021).
• Social factors: Legatum index of social capital for the year 2018, obtained from the World Bank database (Inglehart et al., 2021).
• Cultural factors: Scores for Hofstede’s six dimensions of national culture – power distance, individualism vs. collectivism, masculinity vs. femininity, uncertainty avoidance, long-term orientation, and indulgence vs. restraint, compiled in the year 2010 and updated with World Values Survey data from the year 2014.
• Religious and spiritual factors: a composite measure of religiosity (affiliation, belief, practice and subjective importance) based on the most recent Pew Research Center survey (2018)
• Health infrastructure: number of hospital beds per 1,000 population for the year 2019, obtained from the World Bank database (Inglehart et al., 2021).

A complete list of these variables, the rationale for their inclusion, and the data sources for each variable is provided in Table 1 (Gielen et al., 2009; Tanuseputro, 2017; Pew Research Center, 2018; van Wijngaarden et al., 2019; Karumathil and Tripathi, 20202020; Hofstede Insights, 2021; Inglehart et al., 2021; The World Bank, 2021; Tran et al., 2021).

**TABLE 1:** Variables examined in association to national attitudes towards euthanasia in selected cases, with their data sources.

Variable and date of assessment	Rationale for inclusion in analysis	Data source
Gross national income per capita (GNI, Atlas method, 2020)	Positive attitudes towards euthanasia and assisted dying appear to correlate positively with national income (Inglehart et al., 2021)	World Bank database (The World Bank, 2021)
Gini coefficient of economic inequality (2020)	Requests for assisted dying appear to come disproportionately from patients belonging to lower socio-economic strata (Tran et al., 2021)	World Bank database (The World Bank, 2021)
Social capital (Legatum index of social capital) (2018)	Social support may reduce the likelihood of a request for euthanasia or assisted dying (van Wijngaarden et al., 2019)	World Bank database (The World Bank, 2021)
Hospital beds per 1,000 population (2019)	Assisted dying may be seen as a “cost-effective” measure in healthcare systems that are burdened or lack resources (Tanuseputro, 2017)	World Bank database (The World Bank, 2021)
Cultural dimensions (power distance, individualism/collectivism, masculinity/femininity, uncertainty avoidance, long-term orientation, indulgence/restraint (2010–2014)	Cultural values and beliefs, and particularly individualism/collectivism, appear to play a major role in shaping attitudes towards euthanasia and assisted dying. (Karumathil and Tripathi, 20202020)	Hofstede Institute database (Hofstede Insights, 2021)
Religiosity (2018)	Stronger religious beliefs are associated with disapproval of euthanasia or assisted suicide in most countries (Gielen et al., 2009; Inglehart et al., 2021)	Pew Research Center survey (Pew Research Center, 2018)

All variables were tested for normality prior to analysis. Three variables (sex ratio, gross national income and religiosity) showed significant deviations from normality (*p* < 0.05, Shapiro-Wilk test) and were conformed to an approximately Gaussian distribution by taking the natural logarithm of these variables. After these transformations were applied, Pearson’s correlation coefficient (*r)* was used to estimate the possible linear relationship between approval of euthanasia in selected cases and the above variables. If a visual inspection of the scatter plots for these variables suggested a non-linear relationship, the curve estimation function of the Statistical Package for Social Sciences, version 20.0 (SPSS 20.0) was used to assess this possibility. Finally, a stepwise multivariate linear regression analysis was carried out to identify which variables were significantly associated with attitudes towards euthanasia overall.

The following countries were included in the final analysis: Algeria, Azerbaijan, Armenia, Brazil, China, Colombia, Ecuador, Egypt, Georgia, Haiti, Iraq, Kuwait, Lebanon, Libya, Mexico, Netherlands, New Zealand, Pakistan, Peru, the Philippines, Rwanda, Slovenia, South Africa, Sweden, Thailand, Tunisia, Uruguay and Yemen. Euthanasia is legal in only two of these countries (Netherlands and New Zealand), while assisted suicide is still illegal or under debate in all of them (Nath et al., 2021).

A correlation matrix of the variables associated with the dependent variable EU-SELECT is presented in Table 2 **.** It may be observed that a number of variables were significantly associated with EU-SELECT in this sample. EU-SELECT was positively correlated with life expectancy, gross national income, social capital and cultural individualism, while negative correlations were observed for religiosity and for the cultural dimensions of power distance and uncertainty avoidance. The strength of these correlations was in the moderate (0.6 < |*r|* < 0.8) range for social capital and power distance, and in the fair (0.3 < |*r|* < 0.6) range for the other variables. No significant correlation was observed for sex ratio, economic inequality, hospital bed availability, or the other three cultural dimensions.

**TABLE 2:** Correlation matrix of socioeconomic, cultural and religious variables associated with national attitudes towards euthanasia in selected cases.

Var	1 EU-S	2 LE	3 GR (ln)	4 GNI (ln)	5 Gini	6 SC	7 HB	8 PD	9 IC	10 MF	11 UA	12 LTO	13 IR	14 Rel (ln)
1	—	0.38^†^	0.10	0.58^††^	−0.02	0.65^††^	0.13	−0.67^††^	0.56^††^	−0.28	−0.52^††^	0.16	0.38	−0.52^†^
2	*	—	0.03	0.78^††^	−0.37	0.41^†^	0.45^†^	−0.31	0.27	−0.55^††^	−0.15	0.15	0.42	−0.46^†^
3	*	*	—	−0.24	0.23	-0.16	0.11	−0.26	0.13	0.09	0.04	−0.09	0.37	0.16
4	*	*	*	—	−0.16	0.74^††^	0.43^†^	-0.48^†^	0.57^††^	−0.51^††^	-0.28	0.31	0.65^††^	−0.61^††^
5	*	*	*	*	—	0.08	−0.35	-0.02	−0.03	0.59^††^	−0.11	−0.28	0.48^†^	0.15
6	*	*	*	*	*	—	0.00	-0.60^††^	0.62^††^	−0.36	−0.37	0.05	0.58^††^	−0.13
7	*	*	*	*	*	*	—	0.08	0.10	-0.18	0.00	0.60^††^	-0.15	−0.56^††^
8	*	*	*	*	*	*	*	—	−0.73^††^	0.35	0.35	−0.10	-0.41	0.19
9	*	*	*	*	*	*	*	*	—	−0.29	−0.47^†^	0.12	0.46	−0.20
10	*	*	*	*	*	*	*	*	*	—	-0.03	−0.26	−0.08	0.21
11	*	*	*	*	*	*	*	*	*	*	—	−0.28	−0.15	0.51^†^
12	*	*	*	*	*	*	*	*	*	*	*	—	−0.11	−0.70^††^
13	*	*	*	*	*	*	*	*	*	*	*	*	—	−0.08

Abbreviations: EU-S, percentage of sample population approving euthanasia in selected cases; LE, life expectancy; GR, gender ratio (percentage of women in the adult population); GNI, gross national income per capita; Gini, Gini coefficient of economic inequality; SC, legatum index of social capital; HB, hospital beds per 1,000 population; PD, IC, MF, UA, LTO, IR, Hofstede’s cultural indices of power distance, individualism-collectivism, masculinity-femininity, uncertainty avoidance, long-term orientation and indulgence-restraint; Rel, composite score of religious affiliation, belief and practice; ln, natural logarithm.

^*^is a placeholder used in tables involving correlation matrices to indicate that the concerned correlation coefficient is already displayed elsewhere in the table.; ^†^Significant at *p* < 0.05.; ^††^Significant at *p* < 0.01.

The results of a stepwise multivariate linear regression analysis, taking EU-SELECT as the dependent variable and all significantly correlated parameters from the bivariate analyses as independent variables, is presented in Table 3 **.** The final model included only two variables – gross national income and uncertainty avoidance – and explained approximately 58% of the variance in attitudes towards euthanasia (*R* ^2^ = 0.628; adjusted *R* ^2^ = 0.581). In this model, gross national income was positively associated with approval of euthanasia in selected cases, while uncertainty avoidance was negatively associated with it.

**TABLE 3:** Stepwise multivariate linear regression analysis of variables associated with national attitudes towards euthanasia in selected cases.

Variable	Regression coefficient (β)	Significance level	Part correlation	Variance inflation factor (VIF)
Gross national income (ln-transformed)	0.54	0.005	0.50	1.16
Hofstede’s index of cultural avoidance of uncertainty	−0.41	0.023	−0.38	1.16

The dependent variable was percentage of each national sample approving of euthanasia in selected cases (EU-SELECT). Variables excluded from the final model were life expectancy, social capital, cultural power distance, cultural individualism, and composite index of religiosity.

Non-linear curve estimation analyses for all variables possibly associated with EU-SELECT are presented in Table 4. In these analyses, a linear relationship was found to provide the best fit for gross national income, social capital, power distance, and religiosity. On the other hand, there was a better fit for non-linear (quadratic or cubic) models than for a linear relationship for life expectancy, individualism/collectivism, masculinity/femininity, and avoidance of uncertainty. No significant relationship was found regardless of model type for sex ratio, hospital bed strength, long-term orientation, and indulgence/restraint. Apart from masculinity/femininity, all these variables were significantly associated with EU-SELECT in bivariate linear analyses as well.

**TABLE 4:** Non-linear curve estimation analyses of variables possibly associated with national attitudes towards euthanasia in selected cases.

Variable	Best curve fit	Significance level	Percentage of variance explained
Life expectancy	Quadratic	<0.001	0.573
Sex ratio	None	NS	—
Gross national income	Linear	0.001	0.342
Gini coefficient	None	NS	—
Social capital	Linear	0.001	0.416
Power distance	Linear	<0.001	0.443
Individualism/collectivism	Quadratic	0.003	0.408
Masculinity/femininity	Cubic	0.004	0.468
Uncertainty avoidance	Cubic	0.002	0.490
Long-term orientation	None	NS	—
Indulgence/restraint	None	NS	—
Religiosity	Linear	0.011	0.295
Hospital bed strength	None	NS	—

What do these results tell us? Though based on a relatively small number of countries, and not specifically addressing the specific case of dementia, they suggest that economic and cultural factors might play an important role in determining attitudes towards assisted dying, whether through the assistance or direct action of a physician.

Two of these findings stand out as particularly paradoxical. First, a higher gross national income was strongly and positively correlated with societal approval of euthanasia in selected cases, and this association remained significant even after correcting for the influence of other variables. Considering that one of the arguments advanced in favour of PAS is the economic burden faced by caregivers as well as society at large, this finding is unexpected, and suggests that economic hardship or deprivation alone may not significantly influence positive attitudes towards PAS. These results are consistent with those of a similar study examining changes in attitudes towards this practice across countries, which also found a positive correlation between higher national income and approval of euthanasia (Inglehart et al., 2021).

Secondly, social capital was also strongly and positively correlated with approval of euthanasia. As social capital measures “the strength of personal and social relationships, institutional trust, social norms, and civic participation in a country” (Duh-Leong et al., 2021), it would be expected that higher social capital might mitigate against the approval of assisted dying, and would instead favour the provision of community support and social welfare (Rodriguez-Alcalá et al., 2019). A possible explanation for this finding is that societies in which there is marked polarization about issues such as assisted dying are characterized by lower levels of social capital (Rapp, 2016). If this is the case, one would expect the plot of social capital against approval of euthanasia to take on a U-shape, with higher levels of social capital in societies with more uniform attitudes (either positive or negative) towards euthanasia, and lower scores in societies where attitudes are less uniform. This is partly supported by the available data (Table 4).

Certain aspects of culture also appeared to be strongly associated with attitudes towards euthanasia. Power distance, a measure of hierarchy and top-down social structure, was negatively correlated with approval. This finding is easily understood given that societies with a high power distance show higher levels of respect and deference towards elders, who are often the “target population” for physician-assisted dying (Moshe and Gershfeld-Litvin, 2020). Conversely, individualism was associated with approval of euthanasia in selected cases. Individualistic societies value personal responsibility, self-image, and autonomy, and privilege the individual and their immediate social circle over the wider community. Given that one of the major reasons cited for choosing or desiring PAS is to preserve one’s autonomy in the face of impending suffering or death, this association is also understandable. Results consistent with this finding have been obtained from earlier research in Poland, Germany and the United States (Kemmelmeier et al., 2002). Similarly, it has been observed that physicians with authoritarian values – corresponding to a high cultural power distance – are less likely to concur with hypothetical requests for euthanasia in patients with dementia (Richter et al., 2001).

Besides these two cultural dimensions, the dimension of uncertainty avoidance was negatively correlated with approval of euthanasia. Uncertainty avoidance refers to the manner in which a society or culture handles ambiguous or unclear situations; a high score on this dimension indicates a low tolerance of uncertainty, and the existence of beliefs or institutions that attempt to avoid ambiguity and provide unequivocal “answers” or “solutions.” Given the ambiguity and uncertainty that surrounds an issue such as assisted dying (Pullman, 2004; Niebroj et al., 2013), it is natural that societies scoring high on uncertainty avoidance would attempt to resolve this through uniform disapproval.

Finally, religiosity was negatively correlated with approval of euthanasia in specific cases. There is a long-standing condemnation of most or all forms of assisted dying in several global religious traditions, including Orthodox Judaism (Bradley, 2009), Christianity (Baeke et al., 2011), and Islam (Madadin et al., 2020). A recent systematic review of attitudes towards PAS across five world religions found largely negative attitudes in Islamic respondents, variable responses in Christian and Jewish respondents, and limited acceptance in Buddhist respondents. Among Christian and Jewish survey participants, but not among Muslims, acceptance of assisted dying was inversely correlated with measures of religiosity, which is consistent with the findings presented above (Chakraborty et al., 2017).

There are certain inherent limitations in the analysis presented above which must be taken into account when interpreting these results. First, they are based on survey samples which may not be completely representative of the country in question, despite the best efforts of researchers. Second, they attempt to capture attitudes towards a complex ethical situation using simple nominal categories, leading to a loss of nuance. Third, as the number of countries for which data was available is relatively small, it is possible that some of the findings represent accidental positives due to multiple testing. Fourth, as the data for different variables was captured at different points in time, they may not reflect changes in social attitudes or economic circumstances that have occurred subsequently. Fifth, as these findings are based on country-level data, they cannot be extrapolated to individual residents of a given country. Finally, as the analyses presented above are cross-sectional in nature, they cannot account for changes in attitudes, particularly in countries where euthanasia has recently achieved legal approval, or where cases involving euthanasia are being debated in courts of law.

Despite these limitations, this analysis suggests that approval of euthanasia – and, by extension, PAS – may be strongest in societies characterized by a high income, higher social capital, low religiosity, higher cultural individualism, and lower cultural uncertainty avoidance. What is intended here is not to present a comprehensive account of all the social and cultural determinants of such attitudes, but to outline a tentative profile of countries where individuals are likely to approve of euthanasia or assisted dying, in the abstract, for selected cases. It is perhaps significant that the countries in which PAS has been legally approved conform to the above profile. A corollary to this is that societal and legal approval of PAS may not be forthcoming in countries or regions with a different socio-economic, religious or cultural configuration. It follows from this that widespread availability or legalization of PAS – particularly in a debatable or “borderline” case such as dementia - would neither be necessary or desirable at a global or international level. Instead, other countries and cultures might benefit from alternative approaches to alleviate the suffering caused to patients and caregivers by this condition. These approaches could include healthcare-based approaches such as case management (Saragih et al., 2021), community-based interventions aimed at supporting patients and their families (De Luca et al., 2021), and even scientific research into the neurobiology of the most distressing manifestations of dementia (Kobayashi et al., 2021) which could lead to the development of safer and better treatment methods.

## Pitfalls Inherent in the Practice of PAS in the Specific Case of Dementia

The purpose of the foregoing analysis was to highlight the marked cross-national variation in attitudes towards PAS in general, and the sociocultural correlates of these variations. The results obtained with regard to national income appear to contradict the purely economic arguments in favour of this practice. In this, the potential dangers associated with the practice of PAS in the specific case of dementia will be examined from three perspectives: those of the patients themselves, their caregivers, and the healthcare professionals involved in PAS. In making these assessments, it is important to rely on logic, evidence, the principles of medical ethics, and the realities of diverse cultures and value systems outside the small number of countries which have endorsed this practice. Indeed, appeals to emotion or sentimentality may lead to a simplistic attitude of approval towards PAS (Nichols, 2013).

**FIGURE 1:**
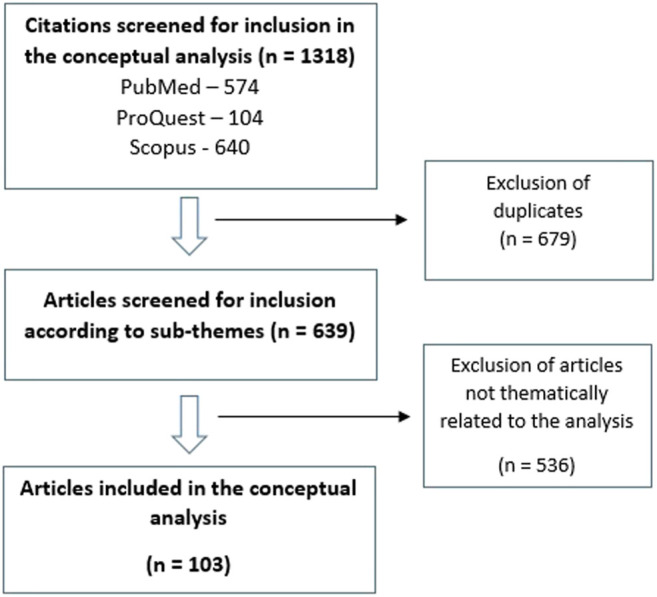
Flow diagram showing the selection of articles for conceptual analysis.

For the purpose of the review and analysis presented below, the PubMed, ProQuest and Scopus literature databases were searched using the broad search terms “dementia” AND either “euthanasia,” “assisted suicide,” “physician-assisted suicide” or “medical assistance in dying.” After removal of duplicates, a total of 642 citations were retrieved via this initial search. Further searches were conducted within these results using the additional search terms “caregiver,” “caregiver burden,” “stress,” “behavioral and psychological symptoms of dementia,” “BPSD,” “economic,” “financial,” “autonomy,” “dignity,” “identity,” “personhood” and “ethics.” By this method, a total of 103 citations were retained (Pereira, 2011; Schuurmans et al., 2021; Kemmelmeier et al., 2002; Bradley, 2009; Baeke et al., 2011; Chakraborty et al., 2017; Madadin et al., 2020; Nichols, 2013; Emanuel et al., 2000; Krag, 2014; Trachtenberg and Manns, 2017; Bilchik, 1996; Lazar and Davenport, 2018; Karrer et al., 2020; Stakišaitis et al., 2019; Finucane et al., 2007; Finucane, 1999; Sachs et al., 2004; Dominguez et al., 2021; Meier, 1997; Liu et al., 2020; Gao et al., 2019; Gilhooly et al., 2016; Watson et al., 2019; Cheng, 2017; Biggs et al., 2019; Fam et al., 2019; Dening et al., 2013; Owen et al., 2001; Cohen-Mansfield and Brill, 2020; Anderson et al., 2019; O'Dwyer et al., 2016; Bravo et al., 2018; Wicher and Meeker, 2012; Stolz et al., 2015; Seike et al., 2021; Kashimura et al., 2021; Zwingmann et al., 2018; Gitlin et al., 2019; von Känel et al., 2019; Zwingmann et al., 2019; Gerk, 2017; Kipke, 2015; Deardorff and Grossberg, 2019; Tiel et al., 2015; Borroni et al., 2008; Kim et al., 2021; Yunusa et al., 2019; Seibert et al., 2021; Dierickx et al., 2017; Scassellati et al., 2020; Hendin et al., 2021; Fornaro et al., 2020; Verhofstadt et al., 2021; Serafini et al., 2016; D'Anci et al., 2019; Buturovic, 2020; Canetto, 2019; Mondragón et al., 2020; Allen, 2020; Rosner and Abramson, 2009; Shannon and Walter, 2004; Alsolamy, 2014; van Wijmen et al., 2015; Brinkman-Stoppelenburg et al., 2020; Mangino et al., 2021; Wardle, 1993; Nicolini, 2021; Mathews et al., 2021; Hertogh, 2009; Jones, 1997; Reagan et al., 2003; Gómez-Vírseda and Gastmans, 2021; Cipriani and Di Fiorino, 2019; Menzel and Steinbock, 2013; Groves, 2006; Fontalis et al., 2018; Gastmans and De Lepeleire, 2010; Ting et al., 2017; Nie et al., 2015; Nakanishi et al., 2021; van der Burg et al., 2019; Largent et al., 2019; Hilliard, 2011; Sharp, 2012; D'cruz, 2021; Cohen-Almagor, 2016; Bolt et al., 2015; Sulmasy et al., 2016; Kenning et al., 2017; Werner et al., 2014; Sulmasy et al., 2018; Dehkhoda et al., 2021; Bravo et al., 2021; Castelli Dransart et al., 2021; Miller et al., 2019; Jongsma et al., 2019; Diehl-Schmid et al., 2017; Cherry, 2003; Johnstone, 2013; Cholbi, 2015; Nicolini et al., 2020; Fuchs and Fuchs, 2021; Huang and Cong, 2021) and these are summarized and analyzed below. This process is depicted in Figure 1.

### Economic Factors

Economic burdens, both those faced by caregivers and by healthcare systems, have been advanced as a justification for PAS. It has already been noted that, paradoxically, approval of PAS in cases such as dementia is higher in high-income countries. Studies of caregivers have also noted that, often, it is not “just” economics that influences attitudes towards PAS. Other factors of equal importance are unmet needs for nursing care, transportation, and domestic assistance, the presence of depressive symptoms in the caregiver, and the caregiver’s perception of the patient’s suffering (Emanuel et al., 2000; Tomlinson et al., 2015). Moreover, even if economic burdens influence a caregiver’s attitudes towards PAS, this need not be interpreted as a reason to broaden access to PAS; it could equally be well seen as a reason to provide economic and logistic assistance to affected families, and to identify and treat depression in caregivers. It has also been noted that, in some cases, those belonging to a higher socio-economic stratum may also be overrepresented among those opting for PAS, again suggesting that simple linear arguments based on caregiver costs do not tell the entire story (Krag, 2014).

The picture that emerges at the level of the healthcare system is different. It has been argued that PAS may lead to substantial savings at the systemic level (Trachtenberg and Manns, 2017); this could lead to a tendency to offer or recommend PAS to patients with dementia as a “cost-effective” measure (Bilchik, 1996). This is a matter of concern, given that cost-driven decisions and policies in healthcare often impose a disproportionate burden on the socially disadvantaged (Lazar and Davenport, 2018). In dementia, economically-driven systemic decisions appear to act as a barrier to the provision of other specific forms of care, such as nursing interventions (Karrer et al., 2020) and may lead to the excessive use of other forms of treatment, such as typical antipsychotics (Stakišaitis et al., 2019) and feeding tubes (Finucane et al., 2007), based on cost considerations rather than evidence. Given this, it is plausible that economic considerations may lead to the incentivization of PAS for patients with dementia, regardless of the best interests of the patient or their caregivers (Finucane, 1999; Sachs et al., 2004). This danger may be especially acute in low- and middle-income countries, where rapid increases in the elderly population and the absence of a social welfare “safety net” may further contribute to such incentivization (Dominguez et al., 2021). Though economic considerations are important in the efficient running of healthcare systems, the interests of the patient should not be subordinated to them (Meier, 1997). In this context, it is also worth discussing the argument made by Krag (Krag, 2014) at more length. His paper is a response to the argument that assisted dying should not be denied to the marginalized groups because this represents a form of “paternalism.” His analysis is based on the fact that paradoxically, rich male individuals in developed countries, who are considered to have greater social power and autonomy, may be equally or even more vulnerable to the threats posed by liberal access to euthanasia or PAS because of culturally conditioned “social messages” that are peculiar to Western countries. His conclusion is that, given that even this “non-marginalized” group is likely to be at risk of the abuse or inappropriate use of PAS, continued restrictions on this practice represent the most prudent course of action. It is of course possible that Krag’s argument would be more applicable to developed and industrialized nations, while concerns related to misuse of PAS in vulnerable groups may be more applicable in lower-income nations with greater economic inequality and resource scarcity. In either case, these arguments favour a more restrictive approach towards PAS.

### Caregiver Burden

As briefly mentioned in the previous section, difficulties faced by caregivers are an important factor driving attitudes towards PAS in patients with dementia. These factors include stress (Liu et al., 2020), sleep disruption (Gao et al., 2019), physical health problems (Gilhooly et al., 2016), syndromal or subsyndromal depression and anxiety (Watson et al., 2019), economic difficulties (Cheng, 2017), and, in some cultures, the stigma attached to a diagnosis of dementia in a family member (Biggs et al., 2019). Though these problems exist globally, they may be particularly acute in low- and middle-income countries where resources for caregivers are limited (Fam et al., 2019). While many of these problems are related to the behavioural problems exhibited by patients with dementia, discussed in the next section, others are not directly correlated with the presence and severity of these behaviours. An argument often advanced in this context is that PAS may be desired by caregivers facing intolerable burdens of this sort, and that therefore it should be made available as a legal option (Tomlinson et al., 2015; Jakhar et al., 2020). However, examination of the responses given by caregivers in such situations reveals a more complex picture. While 40% of carers in a small sample from a developed country did contemplate the possibility of PAS, the same respondents also mentioned that they would prefer optimal end-of-life care to PAS. In the overall sample, a recurrent theme was that if the health care and social care systems were more attuned to the needs of people with dementia and their caregivers, their overall burden would be minimized and they would be less likely to consider PAS favourably (Dening et al., 2013). A similar study compared reactions to death in caregivers of patients with Alzheimer’s disease from different ethnic groups. These researchers observed that African-American caregivers were less likely than White caregivers to approve of even passive forms of “assisted death”, such as withholding care towards the end of life (Owen et al., 2001). Likewise, a more recent study presented Israeli caregivers who had provided end-of-life care to a relative with two end-of-life scenarios, one related to advanced dementia and one to physical disability. Responses to the dementia scenario were ambivalent, with only 48% of the sample (40 of 83 subjects) expressing a clear preference for PAS (Cohen-Mansfield and Brill, 2020). A qualitative analysis of blog posts made by dementia caregivers found a similar lack of uniformity – while themes related to death (*n* = 73), deterioration (*n* = 57), hospice care (*n* = 57) and decision-making (*n* = 41) were expressed across several posts, explicit references to euthanasia or PAS were much less common (*n* = 12); even references to suicidal ideation on the part of the caregiver were relatively more frequent (*n* = 15) (Anderson et al., 2019). In a similar vein, a study assessing overt homicidal ideation in a sample of 21 carers of patients with dementia found that only two subjects overtly expressed such ideation, while four expressed a wish for the patient to die with no homicidal intent. A further four subjects reported aggressive behaviour, verbal or physical, towards the patient, but no wish for the patient to die or be killed. The majority of caregivers (11/21, 52.4%) denied any such ideations or behaviour (O'Dwyer et al., 2016). All the above studies were conducted in regions where PAS is illegal. On the other hand, in a study conducted in a region where PAS had recently been legalized, 68% of caregivers were willing to consider PAS for a relative with advanced Alzheimer’s, with the figure rising to 91% for cases of Alzheimer’s considered to be “terminal” (Bravo et al., 2018).

The significant discrepancy between these results suggests that legalization of PAS may produce significant shifts in the attitudes of caregivers towards this practice, regardless of their earlier attitudes; moreover, such attitudes and shifts are unlikely to be uniform, and may be crucially influenced by variables such as sex and ethnicity (Owen et al., 2001; Wicher and Meeker, 2012; Stolz et al., 2015; Cohen-Mansfield and Brill, 2020) as well as by individual political and religious beliefs (Kemmelmeier et al., 2002; Richter et al., 2001; O'Dwyer et al., 2016). These are not independent of each other; for example, a survey of African-Americans found that several factors, including their cultural and spiritual values and their attitude towards the healthcare system, influenced their lower preference for euthanasia or PAS (Wicher and Meeker, 2012). It is also important to note that none of these studies examined the effect of crucial confounding variables, such as caregiver depression or physical ill-health, economic difficulties, or patient behavioural problems, on attitudes towards PAS. Given the drastic and “final” nature of PAS as a proposed solution for caregiver burden in dementia, it would be prudent to carefully assess such relationships first, and to consider alternate forms of assistance that do not entail the immediate death of the patient. There is evidence for the effectiveness of several such alternatives, including educational interventions (Seike et al., 2021), interventions aimed at strengthening coping skills (Kashimura et al., 2021), case-based care management (Zwingmann et al., 2018) and community-based services (Gitlin et al., 2019). Other strategies that have empirical or theoretical support, but have not yet been evaluated in controlled trials, include better physical and mental health services for caregivers (von Känel et al., 2019), and the assessment and provision of social, financial and legal support tailored to individual needs (Zwingmann et al., 2019). While such interventions may require more investment in terms of manpower, infrastructure and budgetary allotment than PAS, this is not in itself a reason to reject them or consider them inferior – especially in regions where there are social, cultural or religious factors which lead to disapproval of assisted dying. Basing decisions regarding PAS on the “least expensive” or “most cost-effective” option subordinates the rights of both patients and caregivers to economic factors (Bilchik, 1996; Meier, 1997; Gerk, 2017) and opens the door to various forms of abuse (Kipke, 2015).

### Behavioral and Psychological Symptoms of Dementia (BPSD)

A wide range of problematic behaviours, grouped together under the umbrella term BPSD, can be observed in patients with dementia. These include apathy, depression, agitation, aggression, delusions, hallucinations, sleep disturbances, and behavioural disinhibition (Deardorff and Grossberg, 2019). Some of these symptoms may be associated with particular causes or subtypes of dementia: for example, depression and apathy are common in vascular dementia (Tiel et al., 2015) while hallucinations are common in dementia with Lewy bodies (Borroni et al., 2008). The presence of these symptoms is associated with an increased risk of harm to patients themselves (for example, through wandering away or refusal of food or medications) and their caregivers (for example, in the case of aggression or sexual disinhibition). In addition to impairing the quality of life of both patients and caregivers, some of these symptoms – particularly agitation, aggression and hallucinations – are associated with a marked elevation in the burden faced by caregivers (Kim et al., 2021). Though such symptoms are conventionally treated with medications such as atypical antipsychotics and antidepressants, their efficacy is modest and their use is often limited by adverse drug reactions (Yunusa et al., 2019; Seibert et al., 2021). For these reasons, BPSD is sometimes cited as an “indication,” or at least as a contributory factor, for the approval of PAS in patients with moderate or severe dementia (Dierickx et al., 2017).

However, there are certain problems with this line of argumentation. First, though currently available therapies for BPSD have significant limitations, this may not be the case in the future. Basic research is beginning to elucidate the molecular mechanisms associated with specific types of BPSD (Scassellati et al., 2020; Degawa et al., 2021; Kobayashi et al., 2021); novel therapeutic strategies are being developed and evaluated (Magierski et al., 2020); and in some cases, non-pharmacological strategies may also be safe and effective (Abraha et al., 2017; Wang et al., 2019). An undue emphasis on PAS would tend to have a “chilling effect” on such lines of research and reduce funding for them, potentially depriving patients of effective alternatives or even of adequate palliative care (Hendin et al., 2021). Second, the presence of treatment-resistant behavioural symptoms is not unique to dementia, but is observed in several neuropsychiatric conditions, including traumatic brain injury (Rahmani et al., 2021), schizophrenia (Campana et al., 2021), and mood disorders (Fekadu et al., 2009; Fornaro et al., 2020). If the presence of these symptoms in dementia is considered a sufficient indication for PAS, this opens the door to the approval of PAS in patients with any severe or resistant mental illness or behavioural disorder; this has already occurred in some countries where PAS has been legalized (Dierickx et al., 2017; Verhofstadt et al., 2021). From an ethical perspective, this would represent a significant paradigm shift from existing standards of care in neuropsychiatry, where suicide is seen as something to be prevented rather than permitted under supervision (Serafini et al., 2016; D'Anci et al., 2019); this could also lead to a “slippery slope” phenomenon where PAS is seen as the simplest or most cost-effective intervention for any difficult-to-treat neuropsychiatric disorder, particularly in vulnerable populations. This would further erode trust in the healthcare system and impede care among patients with such disorders (Buturovic, 2020). Such a concern is not merely theoretical; there is already evidence from a Belgian series that women are far more likely to undergo PAS for dementia or mood disorders than men (Dierickx et al., 2017). In the case of PAS for women, the analysis by Canetto (Canetto, 2019) is particularly noteworthy. This model posits that White women are especially likely to both advocate for and opt for PAS, and that this arises from a unique combination of privilege and disadvantage. On the one hand, White women are more likely than ethnic minority women to trust the healthcare system (Wicher and Meeker, 2012); on the other hand, they are exposed to disadvantages in terms of economic status, access to palliative care, and cultural ideas of femininity as “self-sacrificing.” These factors interact with aggressive messaging from physicians, experts and the media about the “desirability” and “dignity” associated with PAS. As with Krag’s (Krag, 2014) analysis of a similar situation in high-income men, such considerations suggest that, depending on social and cultural contexts, groups that are thought of as “privileged” may actually be paradoxically vulnerable to an indiscriminate adoption of euthanasia or PAS.

### Issues Specific to Advanced Dementia

Advocates of PAS in dementia could credibly respond to the three preceding sections by suggesting that the practice should be confined to patients with severe or “terminal” dementia, where the patient’s life expectancy is already low and there is little or no scope for improvement (Mondragón et al., 2020). The typical case scenario discussed in this context is that of a patient with advanced dementia who has difficulties in feeding himself, has limited or no mobility, and has developed (or is at risk of developing) complications such as decubitus ulcers or aspiration pneumonia (Cohen-Mansfield and Brill, 2020). In discussing such scenarios, an important distinction needs to be made between passive acts (refusal or denial of care) and active assistance on the part of the physician, such as provision or administration of a lethal drug, as would occur in PAS (Allen, 2020). In the former care, a further distinction can be profitably made between life-sustaining, basic forms of care, such as nutrition and hydration, and “heroic” forms of care, such as aggressive pharmacological treatment or repeated attempts at resuscitation. While the former is considered a form of PAS in several religious traditions and therefore unacceptable (Shannon and Walter, 2004; Rosner and Abramson, 2009; Alsolamy, 2014), the latter would be considered permissible, and could addressed through advance care planning (van Wijmen et al., 2015).

If these cases are excluded and only assisted dying (euthanasia or PAS) is taken into consideration, a different picture emerges, with significant implications for the legalization and implementation of this practice. First, even in countries where PAS is legal for advanced dementia, there is significant ambivalence among both physicians and caregivers. For example, in a survey of Dutch physicians, 53% reported a significant emotional burden when faced with dementia-related PAS requests; 47% had difficulty in evaluating the competency of the patient with reference to informed consent; and 43% reported feeling pressurized by caregivers into approving the request (Schuurmans et al., 2021). Similarly, a sample of the Dutch general public, 40% of respondents considered PAS unacceptable even in advanced dementia; disapproval was stronger in older subjects and in those with higher self-reported religiosity (Brinkman-Stoppelenburg et al., 2020). It is also possible that individuals may express approval of PAS as an abstract notion, but be more disapproving when presented with concrete cases. This was observed in a study of the general public in the United States, where over 54% of respondents expressed approval of PAS for dementia initially, but only 21–40% continued to express approval when provided with specific scenarios (Mangino et al., 2021). The results of these surveys suggests that significant conflicts of interest could arise in this setting; though the Schuurmans et al. (2021) study raises the possibility of physicians feeling “pressured” by family members (Wardle, 1993), it is equally conceivable that caregivers could feel “pressured” for economic, social or other systemic reasons (Kemmelmeier et al., 2002). This could lead to consent or approval being given under duress, and thus being of limited validity. Second, though the “advanced” or “severe” nature of dementia may be evident in certain cases, there are others where it may be difficult to distinguish between “early” and “late” or “moderate” and “severe” cases (Nicolini, 2021). Third, there is evidence that the availability of PAS may compromise the general standard of medical care offered to such patients (Mathews et al., 2021). Fourth, it is also possible that patients with advanced dementia may be partially or wholly unaware of “suffering” as we understand it, and that attempts to frame the debate in these terms may reflect the projection of caregivers’ or physicians’ opinions rather than the patient’s actual situation (Hertogh, 2009). It can be argued, on the basis of these factors, that it would be ethically imprudent to advocate for a procedure that can be misused or inappropriately applied. What is needed, instead, is the identification a “middle position” that recognizes the futility of aggressive or “heroic” treatments in advanced dementia, while avoiding the pitfalls associated with euthanasia or PAS (Jones, 1997; Hendin et al., 2021).

### Patient Autonomy, Dignity and the Right to Die

In view of the cognitive deterioration that inevitably accompanies dementia, the last argument made in this context centers on the primacy of patient autonomy and of the patient’s wishes. It is argued that, given the loss of autonomy that is entailed by cognitive decline, patients should have the right to choose PAS *via* advance directive prior to the onset of such decline. This is seen as a means of preserving their dignity when faced with disintegration of their identity and autonomy (Reagan et al., 2003; Gómez-Vírseda and Gastmans, 2021). Further, it is argued that since informed consent may be impossible once this disintegration has occurred, such an option should not be restricted only to “advanced” cases (Cipriani and Di Fiorino, 2019), and should be included in advance directives (Menzel and Steinbock, 2013) under the principle of “precedent autonomy” (Groves, 2006). This argument is, in a sense, complementary to the previous one, as it sees the suffering and loss of dignity seen in advanced dementia as being “preventable” through PAS (Gómez-Vírseda and Gastmans, 2021).

Though this argument may be more ethically sound than the previous one, as it involves informed consent from patients themselves, it still entails certain difficulties. First, the notion of patient autonomy as a “fundamental principle” is specific to a certain school of Western ethical thought (Cipriani and Di Fiorino, 2019). It can be argued that this principle is not absolute with regards to end-of-life decisions (Fontalis et al., 2018), and that, when viewed from a different philosophical perspective, euthanasia or PAS may not be an ethically viable response to a “fear of disintegration” (Gastmans and De Lepeleire, 2010). Likewise, autonomy-based arguments may be rejected in non-Western cultures, particularly those in which filial piety and respect for the elderly are valued (Ting et al., 2017), or where autonomy is subordinate to community-based values (Nie et al., 2015). Second, it is difficult to evaluate whether an individual patient’s wish for PAS is truly “autonomous” or is the result of coercion, either by family members, by professionals, or by broader socio-economic pressures. This is vividly illustrated by a recent survey of dementia specialists, which found that one or more of these concerns was raised by 63% of respondents (Nakanishi et al., 2021). Third, with advances in the identification of “pre-dementia” through biomarker techniques, the possibility of PAS in pre-symptomatic individuals has been seriously considered by some authors (van der Burg et al., 2019). However, a survey of individuals with elevated amyloid-beta, a putative biomarker for Alzheimer’s risk, found that only 20% of respondents would consider PAS in this context, suggesting that there is a mismatch between the theoretical values espoused in the literature and the actual wishes of patients in this context (Largent et al., 2019). Fourth, reducing an individual’s worth or reason for living to their cognitive capacity is an example of utilitarian thought (Hilliard, 2011), and could lead to the extension of this practice to those with severe mental disability of any sort, as well as to the advocacy of non-voluntary euthanasia on utilitarian or economic principles (Sharp, 2012). Fifth, there are certain dangers in relying on an advance directive in such cases, because an individual’s wishes may vary over time: a patient with early dementia might express a wish for PAS due to psychosocial factors (such as depression or economic hardship) at one point in time, but express a different attitude if such problems are ameliorated (D’cruz, 2021). These conceptual and practical difficulties suggest that the case for PAS in “early” dementia is far from straightforward, and leaves open the possibility of “secondary gain” in which “societally driven” or “coerced” PAS becomes more frequent in this population (Hilliard, 2011; Nakanishi et al., 2021).

## Additional Arguments That Require Consideration When Considering PAS in Dementia

Besides the above factors, which have been the most extensively discussed in the literature, several authors have raised concerns related to the practice of PAS in general, and in this population in particular. Four of these were raised in a recent review (Cohen-Almagor, 2016). First, advocacy for PAS by healthcare professionals involved in dementia care could be seen as violating the principle of beneficience, which is one of the pillars of medical ethics. Second, dementia is generally not a condition associated with severe, intractable pain or other forms of suffering that are seen other terminal illnesses; thus, it would be fallacious to argue for PAS on the basis of “suffering” in these patients. Third, as was mentioned in the previous section, reducing the worth of a patient’s life to their cognitive capacities alone poses certain problems; patients with dementia may continue to live in an “experiential” way even if severely cognitively impaired. Fourth, the finality of ending a patient’s life means that any decisions made in this regard by a third party are problematic, and caution is necessary. These four considerations are not purely theoretical, as can be seen from the results of the surveys discussed earlier, which indicate marked ambivalence regarding PAS on the part of both healthcare professionals and elderly individuals themselves (Dening et al., 2013; Bolt et al., 2015; Schuurmans et al., 2021).

Related to these arguments, Sulmasy et al. have pointed out that the endorsement of PAS creates a fundamental conflict between a physician’s role as providing care to the vulnerable, and their participation in a destructive act (Sulmasy et al., 2016). This could compromise professional integrity and, over time, lead to ambiguities or even erosion of trust in doctor-patient relationships and the healthcare system among patients and their caregivers. This is particularly important in the case of dementia, where there are already significant barriers to care (Werner et al., 2014; Kenning et al., 2017). Elaborating on these points in a further review (Sulmasy et al., 2018), the same author draws on the same argument, and further adduces arguments that have been discussed earlier in this paper, such as the limits of autonomy, the distinction between active killing and passive denial of particular treatments, the social ramifications of suicide and assisted suicide, and the possibility of a “slippery slope” characterized by “incremental extension.” Based on these, he concludes that the medical profession should continue its opposition to PAS on both prudential and ethical grounds. A different but related argument was offered by Kipke (2015), who pointed out that, once one endorses PAS, there are no coherent ethical objections to the provision of assisted suicide outside the healthcare system, including the commercialization of this practice; in other words, permitting PAS in a medical setting could eventually lead to the implementation of this practice on a for-profit basis.

In addition, there is the argument from the lack of consensus amongst medical professionals and the general public. Consensus among experts regarding the value of PAS for dementia, and the feasibility of safeguards against abuse of this practice, is relatively easy to obtain (Dehkhoda et al., 2021). On the other hand, disagreements and disapprovals of this practice among physicians and the general public, who are more aware of concrete realities and of the illusory nature of these safeguards, have been well documented across several settings and countries (Owen et al., 2001; Pereira, 2011; Wicher and Meeker, 2012; Alsolamy, 2014; van Wijmen et al., 2015; Cohen-Mansfield and Brill, 2020; Bravo et al., 2021; Schuurmans et al., 2021). A recent systematic review of studies of older adults underlined this lack of consensus, with only a minority consistently expressing acceptance of PAS, and a significant influence of age, religiosity, education and socio-economic status (Castelli Dransart et al., 2021).

An additional argument based on caution comes from concerns about the failure of safeguards (Pereira, 2011). Analyses of real-world cases reveal the very real potential of ethical violations, as in a recent case where the final decision regarding euthanasia in a patient with dementia was taken by physicians, despite the patient’s apparent ambivalence, and included the surreptitious administration of a sedative to the patient prior to euthanasia (Jongsma et al., 2019; Miller et al., 2019). It is also worth noting that while dementia is not consistently associated with completed suicide, rates of assisted dying in this population have been noted to increase when it is legally permitted (Diehl-Schmid et al., 2017); this phenomenon is reminiscent of the increased suicide rates seen in countries or cultures where access to means of suicide is easier (Sarchiapone et al., 2011).

Finally, as Johnstone (2013) has pointed out, the use of dementia in public debates over assisted dying has led to the adoption of problematic imagery and metaphors to describe dementia. This could lead to the further stigmatization of patients with this disorder, and to an undue emphasis on euthanasia or PAS as the “solution” for those suffering from this illness.

Beyond a biomedical or bioethical framework, there are significant objections to the practice of PAS, both in general and with reference to dementia, in many of the world’s religious traditions (Chakraborty et al., 2017). Though faith-based arguments are often critiqued by those who do not share such beliefs (D’cruz, 2021), they should not be discarded outright. These traditions, even if viewed from a purely naturalistic perspective, are the result of centuries of tradition aimed at safeguarding communities and ensuring justice (Cherry, 2003) and share with medical ethics a desire to safeguard human life and dignity (Fuchs and Fuchs, 2021).

The above review necessarily contains certain limitations, based as it is on a combination of findings from observational studies and analyses of ethical arguments. First, as noted above, responses given by study subjects in surveys are crucially influenced by methodological issues, such as the manner in which a question is framed; thus, some of the lack of uniformity in results may reflect the influence of these factors. Second, as this field of debate is still relatively young, and societal attitudes towards this practice are changing rapidly in some parts of the world, a cross-sectional review of this sort may fail to identify significant shifts in attitudes towards PAS (Nicolini et al., 2020). Finally, due to the heterogeneity and semi-qualitative nature of the material being considered, a formal systematic review or meta-analysis was not possible. These limitation are, to a certain degree, inherent to the complex nature of the question being addressed in this paper.

## Conclusion

A careful examination of existing global survey data and its correlates, as well as of surveys of patients, caregivers and physicians and of ethical arguments for and against PAS in dementia, reveals a picture that is far from cut-and-dried. Favourable attitudes towards PAS appear to be strongly conditioned by cultural and economic conditions and are far from universal. Elderly people, their caregivers, and healthcare professionals all experience significant ambivalence around the issue, and have flagged several areas where abuse of PAS is a real possibility. Conventional arguments in favour of this practice in dementia each have their own limitations, and in each case, alternatives to PAS are both conceivable and feasible in principle. Finally, patients, caregivers and healthcare professionals may all experience significant duress with reference to PAS, due to conflicting interests, physical and mental health status, and social and economic adversity. In the face of this accumulated evidence, it is far from clear that the widespread legalization of PAS is either necessary or desirable. The principle of “first do no harm” should be kept in mind when approaching this issue; it should be understood from the foregoing discussion that “harm” in this case applies not only to patients or physicians but to the physician-patient relationship, the healthcare system, and even society at large. It is essential to avoid a situation where patients or caregivers are made to believe that dementia is associated with a “duty to die” (Cholbi, 2015; Huang and Cong, 2021). What is needed is not advocacy of PAS as a “quick fix” for the complex problems encountered by patients with dementia and their caregivers, but “respecting patients’ humanity and providing them with more care, compassion, and good doctoring.” (Cohen-Almagor, 2016; Hendin et al., 2021), and an attitude of neutrality or passivity on the part of the medical profession is, as Sulmasy et al. (2018) point out, inappropriate in this context.

## References

[B1] Abraha I. Rimland J. M. Trotta F. M. Dell'Aquila G. Cruz-Jentoft A. Petrovic M. (2017). Systematic Review of Systematic Reviews of Non-pharmacological Interventions to Treat Behavioural Disturbances in Older Patients with Dementia. The SENATOR-OnTop Series. BMJ Open 7, e012759. 10.1136/bmjopen-2016-012759 PMC5372076 28302633

[B2] Allen W. (2020). Medical Ethics Issues in Dementia and End of Life. Curr. Psychiatry Rep. 22, 31. 10.1007/s11920-020-01150-7 32388736

[B3] Alsolamy S. (2014). Islamic Views on Artificial Nutrition and Hydration in Terminally Ill Patients. Bioethics 28, 96–99. 10.1111/j.1467-8519.2012.01996.x 22845721

[B4] Anderson J. Eppes A. O’Dwyer S. (2019). “Like Death Is Near”: Expressions of Suicidal and Homicidal Ideation in the Blog Posts of Family Caregivers of People with Dementia. Behav. Sci. 9, 22. 10.3390/bs9030022 PMC6466584 30832390

[B5] Baeke G. Wils J. P. Broeckaert B. (2011). 'We Are (Not) the Master of Our Body': Elderly Jewish Women's Attitudes towards Euthanasia and Assisted Suicide. Ethn. Health 16, 259–278. 10.1080/13557858.2011.573538 21660785

[B6] Biggs S. Carr A. Haapala I. (2019). Dementia as a Source of Social Disadvantage and Exclusion. Australas. J. Ageing 38 Suppl 2, 26–33. 10.1111/ajag.12654 31496064

[B7] Bilchik G. S. (1996). Dollars & Death. Money Changes Everything. Now It's Entering the Debate over the Right to Die-Wwith Explosive Results. Hosp. Health Netw. 70, 18–22. 8994376

[B8] Bolt E. E. Snijdewind M. C. Willems D. L. van der Heide A. Onwuteaka-Philipsen B. D. (2015). Can Physicians Conceive of Performing Euthanasia in Case of Psychiatric Disease, Dementia or Being Tired of Living? J. Med. Ethics 41, 592–598. 10.1136/medethics-2014-102150 25693947

[B9] Borroni B. Agosti C. Padovani A. (2008). Behavioral and Psychological Symptoms in Dementia with Lewy-Bodies (DLB): Frequency and Relationship with Disease Severity and Motor Impairment. Arch. Gerontol. Geriatr. 46, 101–106. 10.1016/j.archger.2007.03.003 17467082

[B10] Bradley C. T. (2009). Roman Catholic Doctrine Guiding End-Of-Life Care: a Summary of the Recent Discourse. J. Palliat. Med. 12, 373–377. 10.1089/jpm.2008.0162 19327075

[B11] Bravo G. Rodrigue C. Arcand M. Downie J. Dubois M. F. Kaasalainen S. (2018). Are Informal Caregivers of Persons with Dementia Open to Extending Medical Aid in Dying to Incompetent Patients? Findings from a Survey Conducted in Quebec, Canada. Alzheimer Dis. Assoc. Disord. 32, 247–254. 10.1097/WAD.0000000000000238 29283927

[B12] Bravo G. Trottier L. Arcand M. (2021). Physicians' Characteristics and Attitudes towards Medically Assisted Dying for Non-competent Patients with Dementia. Can. J. Aging 2021, 1–8. 10.1017/S0714980821000088 34053473

[B13] Brinkman-Stoppelenburg A. Evenblij K. Pasman H. R. W. van Delden J. J. M. Onwuteaka-Philipsen B. D. van der Heide A. (2020). Physicians' and Public Attitudes toward Euthanasia in People with Advanced Dementia. J. Am. Geriatr. Soc. 68, 2319–2328. 10.1111/jgs.16692 32652560 PMC7689700

[B14] Buturovic Z. (2020). Embracing Slippery Slope on Physician-Assisted Suicide and Euthanasia Could Have Significant Unintended Consequences. J. Med. Ethics 472020, 257106089. 10.1136/medethics-2020-106089 32220873

[B15] Campana M. Falkai P. Siskind D. Hasan A. Wagner E. (2021). Characteristics and Definitions of Ultra-treatment-resistant Schizophrenia - A Systematic Review and Meta-Analysis. Schizophr Res. 228, 218–226. 10.1016/j.schres.2020.12.002 33454644

[B16] Canetto S. S. (2019). If Physician-Assisted Suicide Is the Modern Woman's Last Powerful Choice, Why Are White Women its Leading Advocates and Main Users? Prof. Psychol. Res. Pract. 50, 39–50. 10.1037/pro0000210

[B17] Castelli Dransart D. A. Lapierre S. Erlangsen A. Canetto S. S. Heisel M. Draper B. (2021). A Systematic Review of Older Adults' Request for or Attitude toward Euthanasia or Assisted-Suicide. Aging Ment. Health 25, 420–430. 10.1080/13607863.2019.1697201 31818122

[B18] Chakraborty R. El-Jawahri A. R. Litzow M. R. Syrjala K. L. Parnes A. D. Hashmi S. K. (2017). A Systematic Review of Religious Beliefs about Major End-Of-Life Issues in the Five Major World Religions. Palliat. Support. Care 15, 609–622. 10.1017/S1478951516001061 28901283 PMC5865598

[B19] Chambaere K. Bilsen J. Cohen J. Onwuteaka-Philipsen B. D. Mortier F. Deliens L. (2010). Physician-assisted Deaths under the Euthanasia Law in Belgium: a Population-Based Survey. CMAJ 182, 895–901. 10.1503/cmaj.091876 20479044 PMC2882449

[B20] Cheng S. T. (2017). Dementia Caregiver burden: a Research Update and Critical Analysis. Curr. Psychiatry Rep. 19, 64. 10.1007/s11920-017-0818-2 28795386 PMC5550537

[B21] Cherry M. J. (2003). Why Physician-Assisted Suicide Perpetuates the Idolatory of Medicine. Christ Bioeth. 9, 245–271. 10.1076/chbi.9.2.245.30278 15254993

[B22] Cholbi M. (2015). Kant on euthanasia and the duty to die: clearing the air. J. Med. Ethics 41, 607–610. 10.1136/medethics-2013-101781 25246636

[B23] Cipriani G. Di Fiorino M. (2019). Euthanasia and Other End of Life in Patients Suffering from Dementia. Leg. Med. (Tokyo) 40, 54–59. 10.1016/j.legalmed.2019.07.007 31387014

[B24] Cohen-Almagor R. (2016). First Do No Harm: Euthanasia of Patients with Dementia in Belgium. J. Med. Philos. 41, 74–89. 10.1093/jmp/jhv031 26661050 PMC4882626

[B25] Cohen-Mansfield J. Brill S. (2020). After Providing End of Life Care to Relatives, what Care Options Do Family Caregivers Prefer for Themselves? PLoS One 15, e0239423. 10.1371/journal.pone.0239423 32977327 PMC7518928

[B26] D'Anci K. E. Uhl S. Giradi G. Martin C. (2019). Treatments for the Prevention and Management of Suicide: A Systematic Review. Ann. Intern. Med. 171, 334–342. 10.7326/M19-0869 31450239

[B27] D'cruz M. M. (2021). Does Alice Live Here Anymore? Autonomy and Identity in Persons Living and Dying with Dementia. Front. Psychiatry 12, 700567. 10.3389/fpsyt.2021.700567 34366930 PMC8339882

[B28] De Luca R. De Cola M. C. Leonardi S. Portaro S. Naro A. Torrisi M. (2021). How Patients with Mild Dementia Living in a Nursing home Benefit from Dementia Cafés: a Case-Control Study Focusing on Psychological and Behavioural Symptoms and Caregiver burden. Psychogeriatrics 21, 612–617. 10.1111/psyg.12721 34008297

[B29] Deardorff W. J. Grossberg G. T. (2019). Behavioral and Psychological Symptoms in Alzheimer's Dementia and Vascular Dementia. Handb Clin. Neurol. 165, 5–32. 10.1016/B978-0-444-64012-3.00002-2 31727229

[B30] Dees M. K. Vernooij-Dassen M. J. Dekkers W. J. Vissers K. C. van Weel C. (2011). 'Unbearable Suffering': a Qualitative Study on the Perspectives of Patients Who Request Assistance in Dying. J. Med. Ethics 37, 727–734. 10.1136/jme.2011.045492 21947807

[B31] Degawa T. Kawahata I. Izumi H. Shinoda Y. Fukunaga K. (2021). T-type Ca2+ Channel Enhancer SAK3 Administration Improves the BPSD-like Behaviors in AppNL-G-F/NL-G-F Knock-In Mice. J. Pharmacol. Sci. 146, 1–9. 10.1016/j.jphs.2021.02.006 33858649

[B32] Dehkhoda A. Owens R. G. Malpas P. J. (2021). Conceptual Framework for Assisted Dying for Individuals with Dementia: Views of Experts Not Opposed in Principle. Dementia (London) 20, 1058–1079. 10.1177/1471301220922766 32408761

[B33] Dening K. H. Jones L. Sampson E. L. (2013). Preferences for End-Of-Life Care: a Nominal Group Study of People with Dementia and Their Family Carers. Palliat. Med. 27, 409–417. 10.1177/0269216312464094 23128905 PMC3652642

[B34] Deodhar J. K. (2016). End-of-life Care and Psychiatry: Current Trends and Future Directions in India. Mens Sana Monogr. 14, 152–170. 10.4103/0973-1229.193077 28031629 PMC5179614

[B35] Diehl-Schmid J. Jox R. Gauthier S. Belleville S. Racine E. Schüle C. (2017). Suicide and Assisted Dying in Dementia: what We Know and what We Need to Know. A Narrative Literature Review. Int. Psychogeriatr 29, 1247–1259. 10.1017/S1041610217000679 28462742

[B36] Dierickx S. Deliens L. Cohen J. Chambaere K. (2017). Euthanasia for People with Psychiatric Disorders or Dementia in Belgium: Analysis of Officially Reported Cases. BMC Psychiatry 17, 203. 10.1186/s12888-017-1369-0 28641576 PMC5481967

[B37] Dominguez J. Jiloca L. Fowler K. C. De Guzman M. F. Dominguez-Awao J. K. Natividad B. (2021). Dementia Incidence, Burden and Cost of Care: A Filipino Community-Based Study. Front. Public Health 9, 628700. 10.3389/fpubh.2021.628700 34055712 PMC8160123

[B38] Duh-Leong C. Dreyer B. P. Huang T. T. Katzow M. Gross R. S. Fierman A. H. (2021). Social Capital as a Positive Social Determinant of Health: A Narrative Review. Acad. Pediatr. 21, 594–599. 10.1016/j.acap.2020.09.013 33017683 PMC11194101

[B39] Emanuel E. J. Fairclough D. L. Slutsman J. Emanuel L. L. (2000). Understanding Economic and Other Burdens of Terminal Illness: the Experience of Patients and Their Caregivers. Ann. Intern. Med. 132, 451–459. 10.7326/0003-4819-132-6-200003210-00005 10733444

[B40] Emanuel E. J. Onwuteaka-Philipsen B. D. Urwin J. W. Cohen J. (2016). Attitudes and Practices of Euthanasia and Physician-Assisted Suicide in the United States, Canada, and Europe. JAMA 316, 79–90. 10.1001/jama.2016.8499 27380345

[B41] Fam J. Mahendran R. Kua E. H. (2019). Dementia Care in Low and Middle-Income Countries. Curr. Opin. Psychiatry 32, 461–464. 10.1097/YCO.0000000000000523 31082844

[B42] Fekadu A. Wooderson S. C. Markopoulo K. Donaldson C. Papadopoulos A. Cleare A. J. (2009). What Happens to Patients with Treatment-Resistant Depression? A Systematic Review of Medium to Long Term Outcome Studies. J. Affect Disord. 116, 4–11. 10.1016/j.jad.2008.10.014 19007996

[B43] Finucane T. E. Christmas C. Leff B. A. (2007). Tube Feeding in Dementia: How Incentives Undermine Health Care Quality and Patient Safety. J. Am. Med. Dir. Assoc. 8, 205–208. 10.1016/j.jamda.2007.01.007 17498602

[B44] Finucane T. E. (1999). Limiting Life-Sustaining Treatment as a Matter of (Insurance) Policy. J. Am. Geriatr. Soc. 47, 1153–1154. 10.1111/j.1532-5415.1999.tb05245.x 10484263

[B45] Fontalis A. Prousali E. Kulkarni K. (2018). Euthanasia and Assisted Dying: what Is the Current Position and what Are the Key Arguments Informing the Debate? J. R. Soc. Med. 111, 407–413. 10.1177/0141076818803452 30427291 PMC6243437

[B46] Fornaro M. Carvalho A. F. Fusco A. Anastasia A. Solmi M. Berk M. (2020). The Concept and Management of Acute Episodes of Treatment-Resistant Bipolar Disorder: a Systematic Review and Exploratory Meta-Analysis of Randomized Controlled Trials. J. Affect Disord. 276, 970–983. 10.1016/j.jad.2020.07.109 32750614

[B47] Fuchs J. W. Fuchs J. R. (2021). Counteracting Throwaway Culture in Daily Clinical Practice. Linacre Q. 88, 65–70. 10.1177/0024363920936080 33487747 PMC7804503

[B48] Gao C. Chapagain N. Y. Scullin M. K. (2019). Sleep Duration and Sleep Quality in Caregivers of Patients with Dementia: a Systematic Review and Meta-Analysis. JAMA Netw. Open 2, e199891. 10.1001/jamanetworkopen.2019.9891 31441938 PMC6714015

[B49] Gastmans C. De Lepeleire J. (2010). Living to the Bitter End? A Personalist Approach to Euthanasia in Persons with Severe Dementia. Bioethics 24, 78–86. 10.1111/j.1467-8519.2008.00708.x 19222445

[B50] Gerk E. (2017). Following the Money. CMAJ 189, E444. 10.1503/cmaj.732875 28385719 PMC5359097

[B51] Gielen J. van den Branden S. Broeckaert B. (2009). Religion and Nurses' Attitudes to Euthanasia and Physician Assisted Suicide. Nurs. Ethics 16, 303–318. 10.1177/0969733009102692 19372125

[B52] Gilhooly K. J. Gilhooly M. L. Sullivan M. P. McIntyre A. Wilson L. Harding E. (2016). A Meta-Review of Stress, Coping and Interventions in Dementia and Dementia Caregiving. BMC Geriatr. 16, 106. 10.1186/s12877-016-0280-8 27193287 PMC4872341

[B53] Gitlin L. N. Marx K. Scerpella D. Dabelko-Schoeny H. Anderson K. A. Huang J. (2019). Embedding Caregiver Support in Community-Based Services for Older Adults: A Multi-Site Randomized Trial to Test the Adult Day Service Plus Program (ADS Plus). Contemp. Clin. Trials 83, 97–108. 10.1016/j.cct.2019.06.010 31238172 PMC7069225

[B54] Gómez-Vírseda C. Gastmans C. (2021). Euthanasia in Persons with Advanced Dementia: a Dignity-Enhancing Care Approach. J. Med. Ethics 2021, 107308. 10.1136/medethics-2021-107308 34016647

[B55] Groves K. (2006). Justified Paternalism: the Nature of Beneficence in the Care of Dementia Patients. Penn Bioeth. J. 2, 17–20. 17146904

[B56] Hendin H. Hendin J. (2021). “Physician-assisted Suicide and Euthanasia in the Netherlands and Oregon: a Medical and Psychological Perspective,” in Oxford Textbook of Suicidology and Suicide Prevention, Ch. 17. Editor Wasserman D. (London: Oxford Unversity Press), 118–124.

[B57] Hertogh C. M. (2009). The Role of advance Euthanasia Directives as an Aid to Communication and Shared Decision-Making in Dementia. J. Med. Ethics 35, 100–103. 10.1136/jme.2007.024109 19181882

[B58] Hilliard M. T. (2011). Utilitarianism Impacting Care of Those with Disabilities and Those at Life's End. Linacre Q. 78, 59–71. 10.1179/002436311803888474 30082933 PMC6026958

[B59] Hofstede Insights (2021). Country Comparison. Available at: https://www.hofstede-insights.com/country-comparison/ (Accessed 11 11, 2021).

[B60] Huang Y. Cong Y. (2021). Persons with pre-dementia have no Kantian duty to die. Bioethics 35, 438–445. 10.1111/bioe.12865 33683716

[B61] Inglehart R. C. Nash R. Hassan Q. N. Schwartzbaum J. (2021). Attitudes toward Euthanasia: A Longitudinal Analysis of the Role of Economic, Cultural, and Health-Related Factors. J. Pain Symptom Manage. 62, 559–569. 10.1016/j.jpainsymman.2021.01.009 33493587

[B62] Jakhar J. Ambreen S. Prasad S. (2020). Right to life or right to die in advanced dementia: physician-assisted dying. Front. Psychiatry 11, 622446. 10.3389/fpsyt.2020.622446 33551882 PMC7858261

[B63] Johnstone M. J. (2013). Metaphors, Stigma and the 'Alzheimerization' of the Euthanasia Debate. Dementia (London) 12, 377–393. 10.1177/1471301211429168 24336950

[B64] Jones D. G. (1997). Aging, Dementia and Care: Setting Limits on the Allocation of Health Care Resources to the Aged. N. Z. Med. J. 110, 466–468. 9451411

[B65] Jongsma K. R. Kars M. C. van Delden J. J. M. (2019). Dementia and advance Directives: Some Empirical and Normative Concerns. J. Med. Ethics 45, 92–94. 10.1136/medethics-2018-104951 29907577

[B66] Karrer M. Hirt J. Zeller A. Saxer S. (2020). What Hinders and Facilitates the Implementation of Nurse-Led Interventions in Dementia Care? A Scoping Review. BMC Geriatr. 20, 127. 10.1186/s12877-020-01520-z 32264881 PMC7140366

[B67] Karumathil A. A. Tripathi R. (20202020). Culture and Attitudes towards Euthanasia: an Integrative Review. Omega (Westport) 2020, 30222820984655. 10.1177/0030222820984655 33377420

[B68] Kashimura M. Rapaport P. Nomura T. Ishiwata A. Tateno A. Nogami A. (2021). Acceptability and Feasibility of a Japanese Version of STrAtegies for RelaTives (START-J): a Manualized Coping Strategy Program for Family Caregivers of Relatives Living with Dementia. Dementia (London) 20, 985–1004. 10.1177/1471301220919938 32326749

[B69] Kemmelmeier M. Wieczorkowska G. Erb H. P. Burnstein E. (2002). Individualism, Authoritarianism, and Attitudes toward Assisted Death: Cross-Cultural, Cross-Regional, and Experimental Evidence. J. Appl. Soc. Psychol. 32, 60–85. 10.1111/j.1559-1816.2002.tb01420.x 12680373

[B70] Kenning C. Daker-White G. Blakemore A. Panagioti M. Waheed W. (2017). Barriers and Facilitators in Accessing Dementia Care by Ethnic Minority Groups: a Meta-Synthesis of Qualitative Studies. BMC Psychiatry 17, 316. 10.1186/s12888-017-1474-0 28854922 PMC5577676

[B71] Kim B. Noh G. O. Kim K. (2021). Behavioural and Psychological Symptoms of Dementia in Patients with Alzheimer's Disease and Family Caregiver burden: a Path Analysis. BMC Geriatr. 21, 160. 10.1186/s12877-021-02109-w 33663416 PMC7934246

[B72] Kipke R. (2015). Why Not Commercial Assistance for Suicide? on the Question of Argumentative Coherence of Endorsing Assisted Suicide. Bioethics 29, 516–522. 10.1111/bioe.12140 25425401

[B73] Kobayashi N. Shinagawa S. Nagata T. Tagai K. Shimada K. Ishii A. (2021). Blood DNA Methylation Levels in the WNT5A Gene Promoter Region: a Potential Biomarker for Agitation in Subjects with Dementia. J. Alzheimers Dis. 81, 1601–1611. 10.3233/JAD-210078 33967051 PMC8293647

[B74] Krag E. (2014). Rich, white, and Vulnerable: Rethinking Oppressive Socialization in the Euthanasia Debate. J. Med. Philos. 39, 406–429. 10.1093/jmp/jhu026 24973246

[B75] Largent E. A. Terrasse M. Harkins K. Sisti D. A. Sankar P. Karlawish J. (2019). Attitudes toward Physician-Assisted Death from Individuals Who Learn They Have an Alzheimer Disease Biomarker. JAMA Neurol. 76, 864–866. 10.1001/jamaneurol.2019.0797 31034041 PMC6583872

[B76] Lazar M. Davenport L. (2018). Barriers to Health Care Access for Low Income Families: a Review of Literature. J. Community Health Nurs. 35, 28–37. 10.1080/07370016.2018.1404832 29323941

[B77] Liu C. C. Lee C. F. Chang T. Liao J. J. (2020). Exploring the Relationship between the Caregiver's Stress Load and Dementia Patient Behavior: A Case Study of Dementia Specialist Outpatient Data from the Southern Medical Center of Taiwan. Int. J. Environ. Res. Public Health 17, 4989. 10.3390/ijerph17144989 PMC7399981 32664394

[B78] Madadin M. Al Sahwan H. S. Altarouti K. K. Altarouti S. A. Al Eswaikt Z. S. Menezes R. G. (2020). The Islamic Perspective on Physician-Assisted Suicide and Euthanasia. Med. Sci. L. 60, 278–286. 10.1177/0025802420934241 32623956

[B79] Magierski R. Sobow T. Schwertner E. Religa D. (2020). Pharmacotherapy of Behavioral and Psychological Symptoms of Dementia: State of the Art and Future Progress. Front. Pharmacol. 11, 1168. 10.3389/fphar.2020.01168 32848775 PMC7413102

[B80] Mangino D. R. Bernhard T. Wakim P. Kim S. Y. (2021). Assessing Public's Attitudes towards Euthanasia and Assisted Suicide of Persons with Dementia Based on Their Advance Request: An Experimental Survey of US Public. Am. J. Geriatr. Psychiatry 29, 384–394. 10.1016/j.jagp.2020.07.013 32807627 PMC7854974

[B81] Materstvedt L. J. Clark D. Ellershaw J. Førde R. Gravgaard A. M. Müller-Busch H. C. (2003). Euthanasia and Physician-Assisted Suicide: a View from an EAPC Ethics Task Force. Palliat. Med. 17, 97–79. 10.1191/0269216303pm673oa 12701848

[B82] Mathews J. J. Hausner D. Avery J. Hannon B. Zimmermann C. Al-Awamer A. (2021). Impact of Medical Assistance in Dying on Palliative Care: a Qualitative Study. Palliat. Med. 35, 447–454. 10.1177/0269216320968517 33126842

[B83] Meier D. E. (1997). Voiceless and Vulnerable: Dementia Patients without Surrogates in an Era of Capitation. J. Am. Geriatr. Soc. 45, 375–377. 10.1111/j.1532-5415.1997.tb00957.x 9063287

[B84] Menzel P. T. Steinbock B. (2013). Advance Directives, Dementia, and Physician-Assisted Death. J. L. Med Ethics 41, 484–500. 10.1111/jlme.12057 23802899

[B85] Miller D. G. Dresser R. Kim S. Y. H. (2019). Advance Euthanasia Directives: a Controversial Case and its Ethical Implications. J. Med. Ethics 45, 84–89. 10.1136/medethics-2017-104644 29502099 PMC6120810

[B86] Mondragón J. D. Salame L. Kraus A. De Deyn P. P. (2019). Clinical Considerations in Physician-Assisted Death for Probable Alzheimer's Disease: Decision-Making Capacity, Anosognosia, and Suffering. Dement Geriatr. Cogn. Dis. Extra 9, 217–226. 10.1159/000500183 31275347 PMC6600029

[B87] Mondragón J. D. Salame-Khouri L. Kraus-Weisman A. S. De Deyn P. P. (2020). Bioethical Implications of End-Of-Life Decision-Making in Patients with Dementia: a Tale of Two Societies. Monash Bioeth. Rev. 38, 49–67. 10.1007/s40592-020-00112-2 32335862 PMC7205770

[B88] Moshe S. Gershfeld-Litvin A. (2020). Old and Depressed? what We Think about Ending Their Suffering-Attitudes toward Euthanasia for Elderly Suffering from Physical versus Mental Illness. Omega (Westport) 2020, 30222820961241. 10.1177/0030222820961241 32962531

[B89] Mukhopadhyay S. Banerjee D. (2021). Physician Assisted Suicide in Dementia: a Critical Review of Global Evidence and Considerations from India. Asian J. Psychiatry 64, 102802. 10.1016/j.ajp.2021.102802 34388669

[B90] Nakanishi A. Cuthbertson L. Chase J. (2021). Advance Requests for Medical Assistance in Dying in Dementia: a Survey Study of Dementia Care Specialists. Can. Geriatr. J. 24, 82–95. 10.5770/cgj.24.496 34079602 PMC8137455

[B91] Nath U. Regnard C. Lee M. Lloyd K. A. Wiblin L. (2021). Physician-assisted Suicide and Physician-Assisted Euthanasia: Evidence from Abroad and Implications for UK Neurologists. Pract. Neurol. 21, 205–211. 10.1136/practneurol-2020-002811 33850034

[B92] Nichols A. K. (2013). Compassion and Love: the Antidote for Sentimentalism at the End of Life. Linacre Q. 80, 380–386. 10.1179/2050854913Y.0000000009 30083015 PMC6026984

[B93] Nicolini M. E. Kim S. Y. H. Churchill M. E. Gastmans C. (2020). Should Euthanasia and Assisted Suicide for Psychiatric Disorders Be Permitted? A Systematic Review of Reasons. Psychol. Med. 50, 1241–1256. 10.1017/S0033291720001543 32482180

[B94] Nicolini M. E. (2021). Physician Aid in Dying for Dementia: The Problem with the Early vs. Late Disease Stage Distinction. Front. Psychiatry 12, 703709. 10.3389/fpsyt.2021.703709 34646173 PMC8503611

[B95] Nie J. B. Smith K. L. Cong Y. Hu L. Tucker J. D. (2015). Medical Professionalism in China and the United States: a Transcultural Interpretation. J. Clin. Ethics 26, 48–60. 25794294

[B96] Niebroj L. Bargiel-Matusiewicz K. Wilczynska A. (2013). Toward the Clarification of Ideas: Medical Futility, Persistent/obstinate Therapy and Extra/ordinary Means. Adv. Exp. Med. Biol. 755, 349–356. 10.1007/978-94-007-4546-9_44 22826086

[B97] O'Dwyer S. T. Moyle W. Taylor T. Creese J. Zimmer-Gembeck M. J. (2016). Homicidal Ideation in Family Carers of People with Dementia. Aging Ment. Health 20, 1174–1181. 10.1080/13607863.2015.1065793 26189537

[B98] Owen J. E. Goode K. T. Haley W. E. (2001). End of Life Care and Reactions to Death in African-American and white Family Caregivers of Relatives with Alzheimer's Disease. Omega (Westport) 43, 349–361. 10.2190/YH2B-8VVE-LA5A-02R2 12569925

[B99] Pereira J. (2011). Legalizing Euthanasia or Assisted Suicide: the Illusion of Safeguards and Controls. Curr. Oncol. 18, e38–45. 10.3747/co.v18i2.883 21505588 PMC3070710

[B100] Pew Research Center (2018). The Age gap in Religion Around the World. Pew Res. Cent. Available at: https://www.pewresearch.com (Accessed 11 10, 2021).

[B101] Pullman D. (2004). Death, Dignity, and Moral Nonsense. J. Palliat. Care 20, 171–178. 10.1177/082585970402000309 15511036

[B102] Rahmani E. Lemelle T. M. Samarbafzadeh E. Kablinger A. S. (2021). Pharmacological Treatment of Agitation And/or Aggression in Patients with Traumatic Brain Injury: A Systematic Review of Reviews. J. Head Trauma Rehabil. 36, E262–E283. 10.1097/HTR.0000000000000656 33656478

[B103] Rapp C. (2016). Moral Opinion Polarization and the Erosion of Trust. Soc. Sci. Res. 58, 34–45. 10.1016/j.ssresearch.2016.02.008 27194650

[B104] Reagan P. Hurst R. Cook L. Zylicz Z. Otlowski M. Veldink J. H. (2003). Physician-assisted Death: Dying with Dignity? Lancet Neurol. 2, 637–643. 10.1016/s1474-4422(03)00536-2 14505588

[B105] Richter J. Eisemann M. Zgonnikova E. (2001). Doctors' Authoritarianism in End-Of-Life Treatment Decisions. A Comparison between Russia, Sweden and Germany. J. Med. Ethics 27, 186–191. 10.1136/jme.27.3.186 11417027 PMC1733385

[B106] Rodriguez-Alcalá M. E. Qin H. Jeanetta S. (2019). The Role of Acculturation and Social Capital in Access to Health Care: A Meta-Study on Hispanics in the US. J. Community Health 44, 1224–1252. 10.1007/s10900-019-00692-z 31273620

[B107] Rosner F. Abramson N. (2009). Fluids and Nutrition: Perspectives from Jewish Law (Halachah). South. Med. J. 102, 248–250. 10.1097/SMJ.0b013e318197f536 19204613

[B108] Sachs G. A. Shega J. W. Cox-Hayley D. (2004). Barriers to Excellent End-Of-Life Care for Patients with Dementia. J. Gen. Intern. Med. 19, 1057–1063. 10.1111/j.1525-1497.2004.30329.x 15482560 PMC1492583

[B109] Saragih I. D. Tonapa S. I. Lin C. J. Lee B. O. (2021). Effects of Case Management Intervention for People with Dementia and Their Carers: a Systematic Review and Meta-Analysis of Experimental Studies. Int. J. Nurs. Stud. 121, 104012. 10.1016/j.ijnurstu.2021.104012 34265500

[B110] Sarchiapone M. Mandelli L. Iosue M. Andrisano C. Roy A. (2011). Controlling Access to Suicide Means. Int. J. Environ. Res. Public Health 8, 4550–4562. 10.3390/ijerph8124550 22408588 PMC3290984

[B111] Scassellati C. Ciani M. Maj C. Geroldi C. Zanetti O. Gennarelli M. (2020). Behavioral and Psychological Symptoms of Dementia (BPSD): Clinical Characterization and Genetic Correlates in an Italian Alzheimer's Disease Cohort. J. Pers Med. 10, 90. 10.3390/jpm10030090 PMC7563608 32823921

[B112] Schuurmans J. Crol C. Olde Rikkert M. Engels Y. (2021). Dutch GPs' Experience of burden by Euthanasia Requests from People with Dementia: a Quantitative Survey. BJGP Open 5, bjgopen20x101123. 10.3399/bjgpopen20X101123 PMC7960528 33172849

[B113] Seibert M. Mühlbauer V. Holbrook J. Voigt-Radloff S. Brefka S. Dallmeier D. (2021). Efficacy and Safety of Pharmacotherapy for Alzheimer's Disease and for Behavioural and Psychological Symptoms of Dementia in Older Patients with Moderate and Severe Functional Impairments: a Systematic Review of Controlled Trials. Alzheimers Res. Ther. 13, 131. 10.1186/s13195-021-00867-8 34271969 PMC8285815

[B114] Seike A. Sumigaki C. Takeuchi S. Hagihara J. Takeda A. Becker C. (2021). Efficacy of Group-Based Multi-Component Psycho-Education for Caregivers of People with Dementia: a Randomized Controlled Study. Geriatr. Gerontol. Int. 21, 561–567. 10.1111/ggi.14175 33949065

[B115] Serafini G. Calcagno P. Lester D. Girardi P. Amore M. Pompili M. (2016). Suicide Risk in Alzheimer's Disease: A Systematic Review. Curr. Alzheimer Res. 13, 1083–1099. 10.2174/1567205013666160720112608 27449996

[B116] Shannon T. A. Walter J. J. (2004). Implications of the Papal Allocution on Feeding Tubes. Hastings Cent. Rep. 34, 18–20. 10.2307/3528689 15379098

[B117] Sharp R. (2012). The Dangers of Euthanasia and Dementia: How Kantian Thinking Might Be Used to Support Non-voluntary Euthanasia in Cases of Extreme Dementia. Bioethics 26, 231–235. 10.1111/j.1467-8519.2011.01951.x 22571425

[B118] Stakišaitis D. Zamarytė-Sakavičienė K. Lesauskaitė V. Jankūnas R. J. (2019). Off-Label Use of Antipsychotic Agents in Dementia: Evidence for the Revision of the Reimbursement Policy. Ther. Innov. Regul. Sci. 53, 549–553. 10.1177/2168479018795857 30200777

[B119] Stolz E. Burkert N. Großschädl F. Rásky É. Stronegger W. J. Freidl W. (2015). Determinants of Public Attitudes towards Euthanasia in Adults and Physician-Assisted Death in Neonates in Austria: a National Survey. PLoS One 10, e0124320. 10.1371/journal.pone.0124320 25906265 PMC4408035

[B120] Sulmasy D. P. Finlay I. Fitzgerald F. Foley K. Payne R. Siegler M. (2018). Physician-assisted Suicide: Why Neutrality by Organized Medicine Is Neither Neutral Nor Appropriate. J. Gen. Intern. Med. 33, 1394–1399. 10.1007/s11606-018-4424-8 29722005 PMC6082198

[B121] Sulmasy D. P. Travaline J. M. Mitchell L. A. Ely E. W. (2016). Non-faith-based Arguments against Physician-Assisted Suicide and Euthanasia. Linacre Q. 83, 246–257. 10.1080/00243639.2016.1201375 27833206 PMC5102187

[B122] Tanuseputro P. (2017). Medical Aid in Dying: What Matters Most? CMAJ 189, E99–E100. 10.1503/cmaj.161316 28246153 PMC5250514

[B123] The World Bank (2021). World Bank Open Data: Free and Open Access to Global Data. Available at: https://data.worldbank.org/ (Accessed 11 10, 2021).

[B124] Tiel C. Sudo F. K. Alves G. S. Ericeira-Valente L. Moreira D. M. Laks J. (2015). Neuropsychiatric Symptoms in Vascular Cognitive Impairment: a Systematic Review. Dement Neuropsychol. 9, 230–236. 10.1590/1980-57642015DN93000004 29213966 PMC5619363

[B125] Ting P. S. Chen L. Yang W. C. Huang T. S. Wu C. C. Chen Y. Y. (2017). Gender and Age Disparity in the Initiation of Life-Supporting Treatments: a Population-Based Cohort Study. BMC Med. Ethics 18, 62. 10.1186/s12910-017-0222-9 29141641 PMC5688717

[B126] Tomlinson E. Spector A. Nurock S. Stott J. (2015). Euthanasia and Physician-Assisted Suicide in Dementia: a Qualitative Study of the Views of Former Dementia Carers. Palliat. Med. 29, 720–726. 10.1177/0269216315582143 25881624

[B127] Tomlinson E. Stott J. (2015). Assisted Dying in Dementia: a Systematic Review of the International Literature on the Attitudes of Health Professionals, Patients, Carers and the Public, and the Factors Associated with These. Int. J. Geriatr. Psychiatry 30, 10–20. 10.1002/gps.4169 25043718

[B128] Trachtenberg A. J. Manns B. (2017). Cost Analysis of Medical Assistance in Dying in Canada. CMAJ 189, E101–E105. 10.1503/cmaj.160650 28246154 PMC5250515

[B129] Tran M. Honarmand K. Sibbald R. Priestap F. Oczkowski S. Ball I. M. (2021). Socioeconomic Status and Medical Assistance in Dying: A Regional Descriptive Study. J. Palliat. Care 2021, 8258597211053088. 10.1177/08258597211053088 PMC9344489 34747239

[B130] van der Burg S. Schreuder F. H. B. M. Klijn C. J. M. Verbeek M. M. (2019). Valuing Biomarker Diagnostics for Dementia Care: Enhancing the Reflection of Patients, Their Care-Givers and Members of the Wider Public. Med. Health Care Philos. 22, 439–451. 10.1007/s11019-018-09883-2 30680512 PMC6710218

[B131] van Wijmen M. P. Pasman H. R. Widdershoven G. A. Onwuteaka-Philipsen B. D. (2015). Continuing or Forgoing Treatment at the End of Life? Preferences of the General Public and People with an advance Directive. J. Med. Ethics 41, 599–606. 10.1136/medethics-2013-101544 25182697

[B132] van Wijngaarden E. Alma M. The A. M. (2019). 'The Eyes of Others' Are what Really Matters: The Experience of Living with Dementia from an Insider Perspective. PLoS One 14, e0214724. 10.1371/journal.pone.0214724 30943277 PMC6447241

[B133] Verhofstadt M. Audenaert K. Van den Broeck K. Deliens L. Mortier F. Titeca K. (2021). Euthanasia in Adults with Psychiatric Conditions: A Descriptive Study of the Experiences of Belgian Psychiatrists. Sci. Prog. 104, 368504211029775. 10.1177/00368504211029775 34263672 PMC10450708

[B134] Vilela L. P. Caramelli P. (2009). Knowledge of the Definition of Euthanasia: Study with Doctors and Caregivers of Alzheimer's Disease Patients. Rev. Assoc. Med. Bras (1992) 55, 263–267. 10.1590/s0104-42302009000300016 19629343

[B135] von Känel R. Mausbach B. T. Dimsdale J. E. Ziegler M. G. Mills P. J. Allison M. A. (2019). Refining Caregiver Vulnerability for Clinical Practice: Determinants of Self-Rated Health in Spousal Dementia Caregivers. BMC Geriatr. 19, 18. 10.1186/s12877-019-1033-2 30669980 PMC6343283

[B136] Wang G. Albayrak A. van der Cammen T. J. M. (2019). A Systematic Review of Non-pharmacological Interventions for BPSD in Nursing home Residents with Dementia: from a Perspective of Ergonomics. Int. Psychogeriatr 31, 1137–1149. 10.1017/S1041610218001679 30334500

[B137] Wardle L. D. (1993). Conscience Clauses Offer Little protection. Most Are Deficient, and many Have Been Met with Hostile Judicial Interpretations. Health Prog. 74, 79–83. 10127335

[B138] Watson B. Tatangelo G. McCabe M. (2019). Depression and Anxiety Among Partner and Offspring Carers of People with Dementia: a Systematic Review. Gerontologist 59, e597–e610. 10.1093/geront/gny049 29878117

[B139] Werner P. Goldstein D. Karpas D. S. Chan L. Lai C. (2014). Help-seeking for Dementia: a Systematic Review of the Literature. Alzheimer Dis. Assoc. Disord. 28, 299–310. 10.1097/WAD.0000000000000065 25321607

[B140] Wicher C. P. Meeker M. A. (2012). What Influences African American End-Of-Life Preferences? J. Health Care Poor Underserved 23, 28–58. 10.1353/hpu.2012.0027 22643461

[B141] World Values Survey (2021). Wave 6: Results by Country, V20180912. Available at: https://www.worldvaluessurvey.org/wvs.jsp (Accessed 11 11, 2021).

[B142] Yunusa I. Alsumali A. Garba A. E. Regestein Q. R. Eguale T. (2019). Assessment of Reported Comparative Effectiveness and Safety of Atypical Antipsychotics in the Treatment of Behavioral and Psychological Symptoms of Dementia: a Network Meta-Analysis. JAMA Netw. Open 2, e190828. 10.1001/jamanetworkopen.2019.0828 30901041 PMC6583313

[B143] Zwingmann I. Hoffmann W. Michalowsky B. Dreier-Wolfgramm A. Hertel J. Wucherer D. (2018). Supporting Family Dementia Caregivers: Testing the Efficacy of Dementia Care Management on Multifaceted Caregivers' burden. Aging Ment. Health 22, 889–896. 10.1080/13607863.2017.1399341 29156941

[B144] Zwingmann I. Michalowsky B. Esser A. Kaczynski A. Monsees J. Keller A. (2019). Identifying Unmet Needs of Family Dementia Caregivers: Results of the Baseline Assessment of a Cluster-Randomized Controlled Intervention Trial. J. Alzheimers Dis. 67, 527–539. 10.3233/JAD-180244 30584136 PMC6398541

